# Deep learning for detecting prenatal alcohol exposure in pediatric brain MRI: a transfer learning approach with explainability insights

**DOI:** 10.3389/fncom.2024.1434421

**Published:** 2024-08-26

**Authors:** Anik Das, Kaue Duarte, Catherine Lebel, Mariana Bento

**Affiliations:** ^1^Department of Biomedical Engineering, University of Calgary, Calgary, AB, Canada; ^2^Department of Radiology, University of Calgary, Calgary, AB, Canada; ^3^Alberta Children's Hospital Research Institute, Calgary, AB, Canada; ^4^Department of Electrical and Software Engineering, University of Calgary, Calgary, AB, Canada

**Keywords:** prenatal alcohol exposure, deep learning, transfer learning, classification, explainability

## Abstract

Prenatal alcohol exposure (PAE) refers to the exposure of the developing fetus due to alcohol consumption during pregnancy and can have life-long consequences for learning, behavior, and health. Understanding the impact of PAE on the developing brain manifests challenges due to its complex structural and functional attributes, which can be addressed by leveraging machine learning (ML) and deep learning (DL) approaches. While most ML and DL models have been tailored for adult-centric problems, this work focuses on applying DL to detect PAE in the pediatric population. This study integrates the pre-trained simple fully convolutional network (SFCN) as a transfer learning approach for extracting features and a newly trained classifier to distinguish between unexposed and PAE participants based on T1-weighted structural brain magnetic resonance (MR) scans of individuals aged 2–8 years. Among several varying dataset sizes and augmentation strategy during training, the classifier secured the highest sensitivity of 88.47% with 85.04% average accuracy on testing data when considering a balanced dataset with augmentation for both classes. Moreover, we also preliminarily performed explainability analysis using the Grad-CAM method, highlighting various brain regions such as corpus callosum, cerebellum, pons, and white matter as the most important features in the model's decision-making process. Despite the challenges of constructing DL models for pediatric populations due to the brain's rapid development, motion artifacts, and insufficient data, this work highlights the potential of transfer learning in situations where data is limited. Furthermore, this study underscores the importance of preserving a balanced dataset for fair classification and clarifying the rationale behind the model's prediction using explainability analysis.

## 1 Introduction

Prenatal alcohol exposure (PAE) can result in structural brain damage in children, potentially leading to persistent abnormalities in physical, behavioral, and cognitive development along with various post-natal health issues such as fetal alcohol spectrum disorder (FASD) (Larkby and Day, 1997). Early detection of the exposure may allow clinicians to initiate earlier interventions tailored to the specific requirements of the children, guidance on effective parenting strategies, medication to reduce the risk of post-natal disorders, etc. Moreover, an objective marker of alcohol exposure might be crucial in cases where children lack accurate prenatal history, assisting in diagnosis and understanding of potential risks.

Magnetic resonance (MR) imaging has become an effective tool that non-invasively provides numerous structural and functional measures of the brain to study developmental processes and potential abnormalities associated with PAE (Moore and Xia, 2022). Moreover, the integration of machine learning (ML) and deep learning (DL) in MR imaging has enhanced its applicability in terms of supporting clinical diagnosis and decision-making (Rana and Bhushan, 2023).

However, developing ML and DL models is challenging for the pediatric population due to the rapidly developing nature of the brain, and due to motion artifacts that can distort the signal intensities of the scan, resulting in poor acquisition quality. Moreover, there is a significant obstacle in developing DL models because of insufficient training data.

This study aims to incorporate a pre-trained DL model to perform feature extraction followed by training a new classifier to distinguish between unexposed participants and participants with PAE based on T1-weighted structural brain MR scans. A pre-trained feature extractor is utilized as a transfer learning (TL) approach to address the drawbacks of limited data and enhance generalizability by leveraging the knowledge and patterns learned from large, diverse datasets. TL refers to a technique that transfers the knowledge obtained from addressing one problem to another (Raghu et al., 2019). In this study, we considered a particular type of TL that includes learning generic features from a large dataset, which entails employing a pre-trained model on a more minor, targeted dataset to adapt to a new task. In addition, we performed explainability analysis through heatmap visualization to understand the rationale behind the model's prediction, identifying the most relevant brain portions responsible for the predictions that might be crucial for clinical decision-making.

The novelty of this work lies in introducing a pre-trained model to perform feature extraction as a TL technique followed by a preliminary approach to incorporate explainable artificial intelligence (AI) technique (Qian et al., 2023) to visualize the brain portions that are considered the most important features in the model's prediction, supporting the transparency and reliability of the DL approach in PAE analysis. Unlike the majority of experiments focused on adult data, this study contributes to the application of the DL technique tailored to the pediatric population (2–8 years), supported by the TL approach and heatmap visualization that may assist in performing further analysis of the PAE condition.

## 2 Related work

There are relatively few studies using ML and DL techniques to understand brain alterations associated with PAE.

Little and Beaulieu (2020) conducted a study utilizing T1-weighted brain MRI to differentiate children with FASD from controls based on regional brain volumes. Two separate datasets containing MR scans of participants aged 5.2–19.5 years were considered where 87 regional brain volumes were extracted using Freesurfer followed by performing a multivariate support vector machine (SVM) classification model. The model achieved 77% accuracy, 64% sensitivity, and 88% specificity on the test data. Moreover, they separately trained two different models on male and female data where the accuracy was 70 and 67% for male and female groups, respectively. Rodriguez et al. (2021) also considered SVM as the traditional ML approach to classify PAE in rodents using resting-state functional MR imaging (fMRI). Their findings demonstrated the efficacy of SVM in detecting PAE in females, with an accuracy rate of 79.20%, whereas the accuracy on male data was 58.30%. Cortical networks and the hippocampus were implicated in the classification, leveraging SVM weight analysis. Fraize et al. (2023) investigated cerebellar volumetric abnormalities in FASD based on MR data from 89 individuals with FASD and 126 controls aged 6–20 years. The study incorporated a logistic regression classifier that discriminated FASD from controls by considering the allometric scaling relationship between various cerebellar volumes and total brain volume. Their findings advocated that intracerebellar gradient might serve as a potential feature to enhance the model's specificity.

In the recent past, only a few investigations were conducted focusing on deep neural networks in the brain MR-based PAE or FASD study. Duarte et al. (2021) presented an experiment assessing an artificial neural network (ANN) to distinguish children with FASD from controls based on psychometric, saccade eye movement, and diffusion tensor imaging (DTI) data. The study investigated several configurations of ANN with dense layers where the best performance with an 88.46% accuracy was achieved for psychometric data. On the other hand, another configuration of ANN with a feature layer was able to achieve 75% accuracy for DTI data. Their findings showed the potentiality of ANN over traditional ML methods. Instead of using MR modalities, Liu et al. (2023) investigated the correlation between PAE and children's facial shape utilizing 3D facial images of individuals aged from 9 to 13 years. The study employed a convolutional network for non-linear dimensionality reduction. The findings illustrated that facial shape may correlate with low-to-moderate levels of PAE, with lessening trends experienced while children get older.

Prior studies lack the extensive investigation of DL approaches to extract features from brain MR scans to further analyze individuals with PAE. Moreover, there is a lack of clarity regarding the explainability of the classification results, leaving a knowledge gap about the brain regions that are responsible for a particular prediction. Our experiments intended to address these limitations by introducing DL techniques, specifically for the pediatric population, where TL was employed to utilize a pre-trained model for feature extraction followed by classifying between participants with PAE and unexposed and introducing explainability to support the reliability of our proposed DL approach in the PAE study.

## 3 Materials and methods

### 3.1 Dataset

The dataset incorporates 279 publicly available T1-weighted structural brain MR scans from unexposed controls (Reynolds et al., 2020) and 122 MR scans from individuals with PAE, where the T1-weighted scans are not publicly accessible. However, Kar et al. (2021) presented the data collection process for the PAE participants, where the identification of PAE was confirmed through caregiver support groups, early intervention services, and Alberta Children's Services in Alberta, Canada. In this study, an annotated dataset for both groups was incorporated, where the annotation indicated whether each scan belonged to an unexposed control or a PAE participant.

The scans were acquired from both male and female participants aged between 2 and 8 years for unexposed controls and PAE participants aged from 2 to 7 years. Using the Fast Spoiled Gradient Echo (FSPGR) BRAVO sequence with a 32-channel head coil, the General Electric 3T MR750w scanner generated brain MR scans, with a matrix size of 512 × 512 with 210 slices and a field of view of 23.0 cm based on the flip angle = 12^*o*^, TR = 8.23 ms, TE = 3.76 ms, TI = 540 ms, isotropic voxel size = 0.9 × 0.9 × 0.9 *mm*^3^ (Reynolds et al., 2020).

### 3.2 Data preprocessing

Several preprocessing steps were executed, including skull stripping (SS), registration, bias field correction, intensity normalization, and resizing. Based on the findings of our previous study on the efficiency of SS methods for pediatric populations (Das et al., 2023), we utilized FSL-BET (Smith, 2002) and SynthStrip (Hoopes et al., 2022) for SS, where the skull and other non-brain tissues were eliminated. A pediatric-specific atlas was considered to register all scans to a common space using FSL-FLIRT (Jenkinson et al., 2012). The N4 technique (Elyounssi et al., 2023) was implemented with a Python-based package called SimpleITK for bias field correction, where no additional acquisitions were considered for bias estimation. Finally, voxel intensities were standardized within a range of 0–1 using min-max normalization followed by resizing scans to meet the input shape requirement of the deep learning model for feature extraction. Additionally, random rotation was employed using MONAI transform (Cardoso et al., 2022) with the default angle value of 10 degrees across three axes as an augmentation approach to increase the training set volume. The probability value was set to 1, ensuring the rotation of all scans in the training set.

### 3.3 Feature extraction

**Figure 1:**
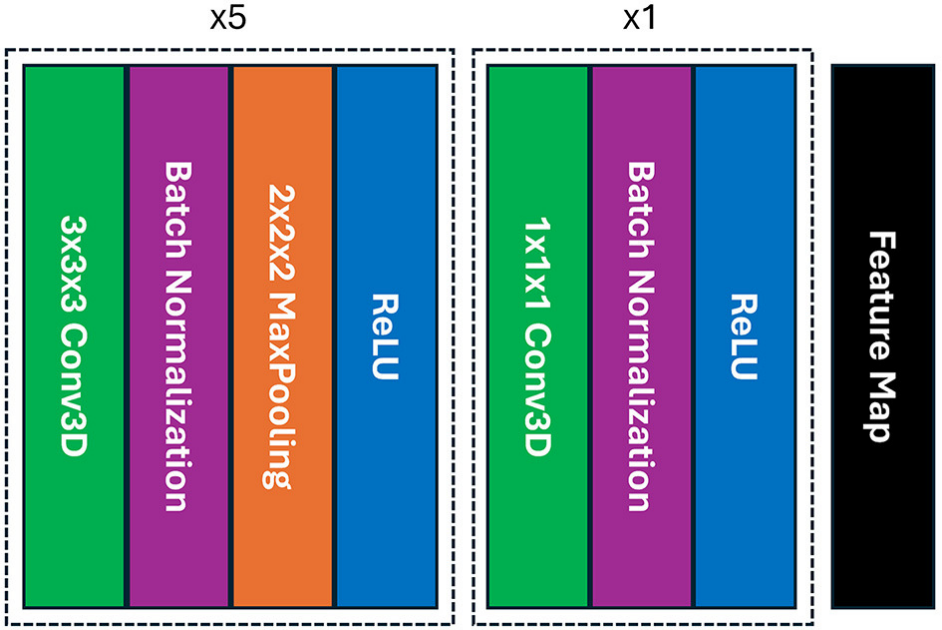
The architecture of the SFCN model's feature extraction part includes six blocks of convolution where each of the first five blocks contains a 3D convolutional layer, a batch normalization, a max pooling, and ReLU activation layers; whereas the max pooling layer is absent in the final block, providing with a feature map.

To perform feature extraction, we utilized the simple fully convolutional network (SFCN) model that was previously trained on the UK-Biobank dataset containing adult data and initially created for precise brain age prediction and biological sex classification in the Predictive Analysis Challenge (PAC) in 2019 (Gong et al., 2021). The SFCN model was considered due to the consistent preprocessing steps between the UK-Biobank dataset and our preprocessing setting, ensuring effective utilization of TL in feature extraction. In this study, we removed the classification layers from the SFCN architecture and only retained the feature extraction part comprising six convolutional blocks, shown in Figure 1. A 3 × 3 × 3 3D convolutional layer, a batch normalization layer, a max pooling layer, and a ReLU activation layer are all present in each of the blocks (Gong et al., 2021). As it moves deeper into the layers, the model's design swiftly shrinks the spatial dimensions of the input data by reducing them to 5 × 6 × 5 in just five blocks. The sixth block skips the max pooling layer and adds a 1 × 1 × 1 3D convolutional layer, which boosts non-linearity while maintaining the spatial dimensions of the feature map. In our study, the pre-trained SFCN model's weight and biases were frozen, thus not altering the feature extraction part, highlighting the TL approach by leveraging the previously trained feature extractor to operate on pediatric data. Each preprocessed brain MR scan was fed into the pre-trained feature extractor, and the result from the final convolutional layer was a feature representation flattened to a one-dimensional vector.

### 3.4 Classification

We added a new classifier layer on top of the pre-trained feature extractor to classify unexposed controls vs. children with PAE. The classifier has 512 fully connected nodes in its first layer. A ReLU activation (Lin and Shen, 2018), a dropout layer with a dropout rate of 0.5, and a final fully connected layer with a single output node with sigmoid activation for binary classification. We utilized binary cross-entropy loss and Adam optimizer (Khan et al., 2021) with a learning rate of 0.001 to train the classifier with 100 epochs. To prevent overfitting, the classifier was trained with 5-fold stratified cross-validation, where the dataset was divided into 80, 10, and 10% for training, validation, and testing, respectively. We performed classification comprising four different cases by considering distinct augmentation approach and varying sizes of the dataset.

**Case I: Imbalanced sataset with augmenting both classes -** In this case, we considered the entire dataset containing 279 unexposed scans and 122 scans of PAE participants, resulting in an imbalance between the two classes. The augmentation was performed on both classes during the classifier training.

**Case II: Imbalanced dataset with augmenting only PAE class -** This case also included the entire dataset to train, validate, and test the model. However, we performed augmentation only on the PAE class, while keeping the unexposed scans without augmentation during training.

**Case III: Balanced dataset with augmenting both classes -** In this case, we constructed a balanced dataset by considering an equal number of scans from both classes which contains randomly selected 122 unexposed scans and 122 scans of PAE. Subsequently, augmentation was employed in both classes.

**Case IV: Balanced dataset with no augmentation -** In this setup, we considered the balanced dataset for the classification. However, no augmentation was introduced to the training dataset.

### 3.5 Explainability analysis

To evaluate the explainability of the feature extraction step, we utilized gradient-weighted class activation mapping (Grad-CAM), a method of visualizing the most relevant areas of a scan that the model considers during prediction as a heatmap (Zhang et al., 2021). The selection of Grad-CAM can be corroborated by its ability to clarify the reasoning behind the complex model's decision-making process without sacrificing accuracy, maintaining a balance between model explainability and high accuracy. The heatmap was constructed by leveraging the gradient flow across the convolutional layer and weighing the feature map using the gradient. Consequently, the heatmap was resized to the input scan size to generate a blended scan by overlaying the heatmap and input MR scan. Grad-CAM based heatmap highlights specific brain regions since it identifies areas where the model's activation is highly influenced by the presence of patterns associated with unexposed and PAE data. The heatmaps were generated using feature maps before performing different classification settings which allowed us to obtain uniform heatmaps across all cases and observe the brain regions that were considered as important features in classification layers.

### 3.6 Statistical analysis

A statistical *t*-test (Moore, 1996) was introduced to observe the significance level of the brain regions that were important in the model's prediction process between unexposed controls and PAE individuals. We considered an independent two-sample *t*-test based on the mean volume of the segmented region to compare the significance of brain area (feature) between the two groups. Finally, *t*-statistic and *p*-value were utilized to understand whether the observed differences were statistically significant or not.

## 4 Results

Table 1 illustrates the classifier's performance on the testing dataset by considering sensitivity, specificity, and average accuracy across 5-fold. In case I, a sensitivity of 69.55% and specificity of 94.73% along with an average accuracy of 86.29% and a standard deviation of 3.64 were experienced, where training data of both classes were augmented. In case II, the model achieved 66.13% sensitivity along with a 95.86% specificity score, resulting in an average accuracy of 85.91% with a deviation of 3.97, when only the PAE data was augmented during training. In case III, the sensitivity and specificity scores were 88.47 and 82.14%, respectively when we considered an equal number of augmented scans during training. In this case, the model secured an average accuracy of 85.04% with a comparatively higher deviation of 5.44 on the testing dataset. However, a slightly higher sensitivity of 88.60% and specificity of 84.34% along with an average accuracy of 86.29% were experienced in case IV, where no augmentation was introduced to the training dataset.

**Table 1:** Comparison of sensitivity, specificity, and average accuracy with standard deviation on the testing dataset for four different cases where case I and case II experienced a higher specificity but a lower sensitivity score. Conversely, cases III and IV exhibited a higher sensitivity and a high average accuracy and deviation.

**Cases**	**Sensitivity (%)**	**Specificity (%)**	**Avg. acc. (%) ± Std. dev**.
I. Imbalanced dataset & Aug. both classes	69.55	94.73	86.29 ± 3.64
II. Imbalanced dataset & Aug. only PAE class	66.13	95.86	85.91 ± 3.97
III. Balanced dataset & Aug. both classes	**88.47**	82.14	**85.04** **±** **5.44**
IV. Balanced dataset & No aug.	88.60	84.34	86.29 ± 4.54

**Figure 2:**
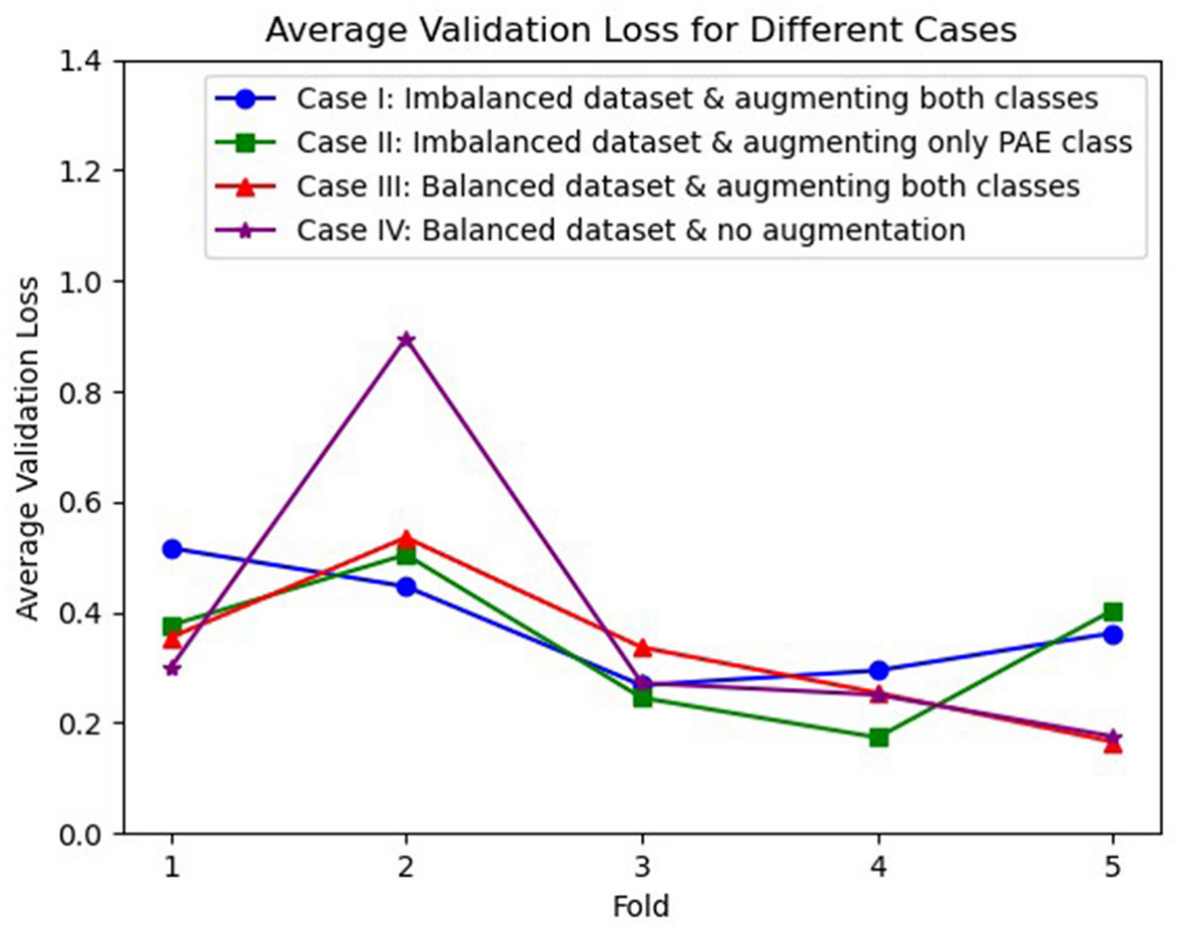
Visualization of average validation loss across 5-fold for three different cases, where case I (blue), case II (green), and case IV (purple) experienced an inconsistent loss whereas case III (red) shows a continuous reduction in loss after the second fold.

The average losses on the validation dataset across 5-fold were also analyzed, illustrated in Figure 2, where we observed an inconsistent decrease in the average loss for case I, case II, and case IV. On the other hand, case III initially experienced a higher loss for the second fold. However, a gradual decrement in the loss was experienced in the following folds.

**Figure 3:**
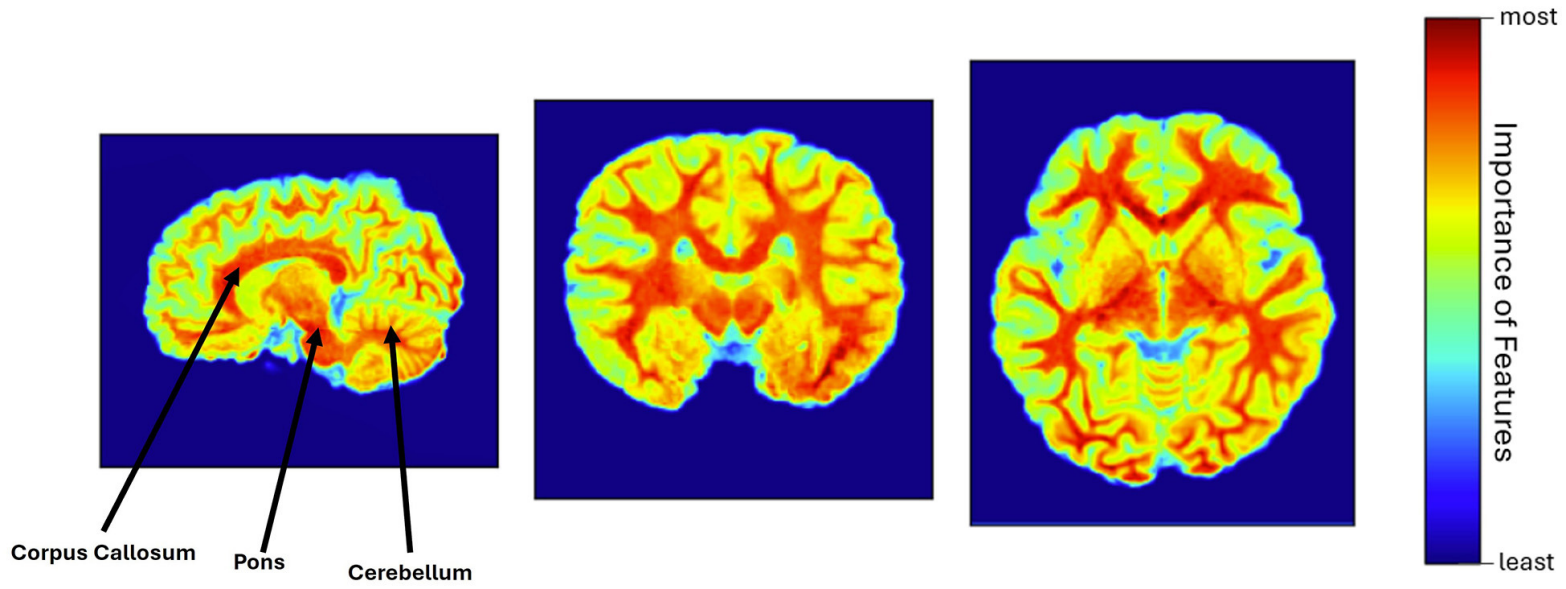
Heatmap visualization using Grad-CAM, where corpus callosum, pons, cerebellum, and white matter were identified as the most relevant features of the brain (warm red color), involved in the model's decision-making process.

The explainability of the feature extractor is shown in Figure 3, by visualizing the heatmap for all scan planes using Grad-CAM. In this study, the pre-trained feature extractor recognized a few brain portions, such as corpus callosum, cerebellum, pons, and brain white matter denoted by warm red color, as the most relevant brain areas for leading the decision-making process and distinguishing between unexposed and participants with PAE.

While brain white matter appeared as one of the important tissues in the model's prediction process, a volume-based statistical *t*-test to compare the significance of the white matter region between the unexposed and PAE groups resulted in a *t*-statistic of −0.73 and a *p*-value of 0.47.

## 5 Discussion

The pediatric brain develops rapidly which creates challenges regarding model sensitivity. Most of the ML and DL tools have been developed for adults. Therefore, introducing DL techniques to work on pediatric problems may have a notable impact on pediatric research and clinical practice. This study aimed to employ DL for the pediatric population which involves constructing a classifier that can distinguish between unexposed and PAE participants based on the features extracted from brain MR scans with a pre-trained model.

The performance of the classifier on the testing dataset in four different contexts revealed that augmenting only the PAE data (case II) resulted in a reduction in sensitivity while preserving a consistent specificity score. This finding highlights the importance of augmenting scans of both classes, since augmenting a single class may not adequately represent the diversity of the dataset.

However, the model's sensitivity may not be enhanced solely by augmenting scans of both classes if the model is trained on a class-imbalanced dataset. Case III and case IV, where an equal number of scans from both classes were considered during training, exhibited higher sensitivity of the model than the other two cases on the testing dataset, emphasizing the importance of addressing the class imbalance in training data.

Moreover, imbalanced class distributions such as in case I and case II may introduce confounding factors in evaluating the model's performance. A high specificity, particularly for the unexposed class, might inflate testing set accuracy. As a result, these metrics may not fairly represent the model's ability to generalize across classes. Conversely, in case III and case IV, the convergence of the sensitivity and specificity along with nearly identical test set accuracy indicates that the model may identify necessary patterns and fairly classify both unexposed and PAE when the dataset is balanced.

Additionally, in case I and case II, despite exhibiting higher specificity and test set accuracy, the sensitivity score (true positive rate) was noticeably lower than case III and case IV which indicates that the model struggled to distinguish PAE scans accurately, resulting in inconsistent performance. On the other hand, the model's consistency on the well-balanced dataset (cases III and IV) can be underscored by its higher sensitivity score along with nearly similar specificity and average accuracy, leading to a superior classification setting compared to the other two cases.

Furthermore, the investigation of average losses across 5-fold on the validation dataset presents insights into the reliability of the model for different experimental settings. The inconsistent trend of average losses for case I and case II suggests potential challenges encountered by the model during the learning phase due to the imbalanced dataset and the absence of augmentation for both classes. On the other hand, although case IV performed slightly better than case III on the test dataset, it is also noticeable that the average validation loss exhibited greater inconsistency when augmentation was absent, referring to the importance of augmentation in a limited data setting to ensure consistent learning across 5-folds. However, despite experiencing an initial higher loss, a decreasing trend in the validation loss for case III indicates a more steady learning process and the reliability of the model in the well-balanced augmented dataset. Although the augmentation might not be representative of higher accuracy, it could play a critical role in reducing the validation loss and ensuring consistent performance.

In this study, different brain regions considered in decision-making were illustrated to provide the model's explainability which may assist clinicians in understanding the rationale behind the model's prediction. This study highlights the involvement of the corpus callosum in the model's prediction, aligning with a prior investigation conducted by Sowell et al. (2001), which also indicated the malformations of the corpus callosum due to alcohol exposure. Moreover, the cerebellum was found in another experiment managed by Bookstein et al. (2006), indicating its vulnerability to the effects of alcohol exposure. The cerebellum was also considered a responsible feature in our experiment. Moreover, this work emphasizes the further detailed investigation of brain white matter since the entire white matter was considered the essential feature in the prediction process. This finding can be corroborated by introducing a prior work conducted by Kar et al. (2021), where they observed a slower white matter development in children with PAE compared to unexposed controls. Furthermore, the pons is taken into account as an important feature during decision-making, suggesting that this brain area may be affected in children due to alcohol exposure. The visual inspection of all heatmaps confirmed the consistent activation patterns of these brain regions.

The consistency of the DL approach can be underscored by its effectiveness in identifying similar features across the dataset. Moreover, aligning our findings with previous literature highlights the significance of explainability analysis and further strengthens the relevance of this study in PAE analysis. Since DL models are usually considered black boxes (Arrieta et al., 2020), leaving an information gap regarding the reasoning behind the model's prediction. In this study, introducing the explainability technique enhanced the transparency, trustworthiness, and accountability of the DL model by enabling insights into its decision-making process. Moreover, understanding brain biomarkers contributing to PAE classification may guide targeted screening and treatment strategies.

While explainability analysis demonstrated various brain regions responsible for the model's prediction process, examining the significance level of those brain regions between unexposed controls and PAE groups might be crucial. However, the absence of an age-specific region-based atlas for the pediatric population (2–8 years old) limits the segmentation of those brain regions. This study generated only the white matter segmentation followed by a volume-based *t*-test, where, a *t*-statistic of −0.73 indicates that the mean white matter volume in the PAE group is slightly lower than in the unexposed control group. However, a higher *p*-value of 0.47 demonstrates that the difference is not statistically significant, mentioning that there is a high probability that any observed difference in white matter volume between the two groups is due to random chance rather than a true underlying difference. The white matter alone may not be the differentiator between the two groups. Instead, other brain regions might need to be considered for more comprehensive analysis.

Although our findings correlate with prior experimental results, limitations still exist in the model's robustness due to limited pediatric brain MR scans. In addition, the applicability of our proposed approach to broader pediatric groups with distinct demographic and clinical characteristics remains unknown. Obtaining more data will allow us to train the model from scratch, which may incorporate more relevant features associated with PAE, potentially leading to improved accuracy in classifying unexposed and PAE participants. Moreover, the study could be extended in the future by incorporating other DL models and explainability techniques to validate the findings and further investigate the significance level of all relevant features between unexposed controls and PAE.

## 6 Conclusion

This study demonstrated the application of DL in the pediatric population (2–8 years) to distinguish between unexposed and PAE participants based on T1-weighted structural brain MR scans. We incorporated the pre-trained SFCN model as a feature extractor, highlighting the TL approach followed by training a classifier on the extracted features. Moreover, this work showcased the rationale behind the model's decision-making process by visualizing heatmaps with the Grad-CAM technique. Overall, we highlighted the potential of DL in analyzing pediatric brains corroborated by TL while dealing with a limited amount of data. This work also emphasized the requirement for a balanced dataset to achieve higher sensitivity (true positive rate), leading to a fair classification (consistency between sensitivity and specificity) for both classes which may ensure the model's generalizability. In addition, incorporating explainability analysis enabled us to observe the most important features considered in the model's decision-making process, supporting the transparency of the model. However, it is essential to note that this analysis has not been examined on other age groups such as infants or teenagers, leaving a knowledge gap regarding its extensive applicability. Our future investigation includes considering other DL models, fine-tuning the model to improve performance, undertaking a more in-depth investigation of explainability to observe the rationale behind the model's prediction with other explainable AI techniques, and performing a comprehensive analysis between two groups using statistical approaches.

## Data Availability

Publicly available datasets were analyzed in this study. This data can be found here: https://osf.io/axz5r/ (T1-weighted MRI).
